# High-Purity, Uniform, and Spherical Hafnium Carbide Nanoparticles Derived from a Novel Amorphous Hafnium-Based Metal–Organic Framework Precursor for the Preparation of High-Performance Ceramics

**DOI:** 10.3390/ma19091754

**Published:** 2026-04-24

**Authors:** Hongzhi Cheng, Jian Gu, Siyuan Kan, Ran Xie, Quan Li, Sinuo Zhang, Junyang Jin, Yang Wang, Jian Yang, Chang-An Wang

**Affiliations:** 1 College of Materials Science and Engineering, Nanjing Tech University, Nanjing 211816, China; 15683037410@163.com (H.C.);; 2 Jiangsu Collaborative Innovation Center for Advanced Inorganic Function Composites, Nanjing 211816, China; 3 Advanced Bio and Healthcare Materials Research Division, Korea Institute of Materials Science, Changwon 51508, Republic of Korea; 4 State Key Lab of New Ceramics and Fine Processing, School of Materials Science and Engineering, Tsinghua University, Beijing 100084, China

**Keywords:** metal-organic frameworks, HfC nanoparticles, low temperature pyrolysis, ultra-high temperature ceramics

## Abstract

**Highlights:**

- A novel amorphous Hf-MOF precursor was synthesized via a simple and efficient method.
- Pure, spherical HfC nanoparticles (44.30 ± 9.63 nm) were synthesized via 1500 °C pyrolysis.
- HfC synthesized at 1700 °C showed low oxygen (0.76%) and near-theoretical carbon (6.42%).
- SPS-sintered HfC achieved 96.7% density and 20.2 GPa hardness, outperforming commercial HfC.

**Abstract:**

A novel amorphous Hf-MOFs precursor was successfully synthesized and converted into HfC nanoparticles via one-step pyrolysis. The effects of metal/ligand molar ratios, solvent types, and pyrolysis temperature were systematically studied. High-purity spherical HfC nanoparticles (44.30 ± 9.63 nm) were obtained at 1500 °C using a 1.5:1 metal/ligand molar ratio with mixed anhydrous ethanol/deionized water solvents. At a pyrolysis temperature of 1700 °C, the as-synthesized HfC nanoparticles possessed an exceptionally low oxygen content of 0.76%, alongside a carbon content of 6.42% that almost perfectly matches the theoretical value of stoichiometric HfC. The formation mechanism involving Hf-O-C coordination and carbothermal reduction was clarified. Additive-free HfC ceramics were fabricated using the as-synthesized HfC nanoparticles via spark plasma sintering (1950 °C, 30 MPa, 20 min). The resulting ceramics exhibited a relative density of 96.7% and a Vickers hardness of 20.2 GPa, both of which are significantly superior to those of ceramics sintered from commercial HfC powders under identical conditions (95.8% and 17.8 GPa, respectively). This work provides a promising and feasible pathway for the preparation of other high-quality ultra-high temperature hafnium-based carbide powders and ceramics.

## 1. Introduction

When the flight speed of a hypersonic vehicle exceeds Mach 5, the intense friction with the atmosphere generates high temperatures exceeding 2000 K in the nose cones and leading edges of the wings. Most materials cannot withstand such extremely high temperatures [1]. A series of materials named as ultra-high temperature ceramics (UHTCs), possessing excellent properties such as high melting point, high mechanical strength and exceptional oxidation resistance, have attracted much attention [2]. However, in practical use under harsh environments, like the classic ceramic materials, the intrinsic low fracture toughness and poor thermal shock resistance of UHTCs still restrict their further application. Previous work has demonstrated that the introduction of continuous fibers as reinforcements into the ceramic matrix can effectively improve these limitations [3,4]. Hence, continuous fiber-reinforced ultra-high temperature ceramic matrix composites (FRUHTCMCs) have become one of the most promising candidate materials for thermal protection components in hypersonic vehicles [5].

Among FRUHTCMCs, HfC-based ceramic matrix composites (HfC-CMCs) are drawing increasing attention due to their low coefficient of thermal expansion, high ablation resistance, and superior mechanical properties [6,7]. Various methods have been employed to synthesize HfC-CMCs, mainly including reactive melt infiltration (RMI) [8], precursor infiltration and pyrolysis (PIP) [9], chemical vapor infiltration (CVI) [10], and slurry infiltration (SI) [11]. Notably, the SI method stands out due to its simpler process, shorter cycle time, lower cost, and minimal damage to fiber reinforcements, making it ideal for synthesizing HfC-CMCs [12]. However, the pore size distribution in fiber preforms spans from tens of nanometers to several hundred micrometers. In order to obtain effective infiltration, the size of ceramic fillers should be at the nanoscale. It should be noted that the nanosized fillers are easy to agglomerate due to their high surface energy, leading to the fabricated slurry with excessively high viscosity and low solid loading, and further hindering its effective penetration into the fiber preform [13]. Meanwhile, our previous work pointed out that ceramic fillers with spherical morphology could reduce interparticle friction, thereby decreasing slurry viscosity and improving infiltration effectiveness [14]. Therefore, high-quality HfC fillers with uniform nanoscale size and spherical morphology are urgently required for synthesizing high-performance HfC-CMCs using SI method.

Common methods, mechanical alloying [15], carbothermal reduction (CTR) [16], liquid-phase precursor conversion (PDC) [17], and sol–gel [18], are frequently employed to synthesize HfC nanoparticles. Mechanical alloying tends to introduce impurities from the milling media, resulting in final samples with low purity. CTR requires a very high reaction temperature, leading to large particle sizes and limited control over morphology. The PDC method is generally used to synthesize HfC nanoparticles containing free carbon and a broad particle size distribution. The sol–gel method for synthesizing HfC nanoparticles involves a lengthy processing period, and controlling the particle morphology remains challenging. Therefore, finding an effective method for synthesizing pure HfC nanoparticles with controllable morphology remains an urgent challenge.

Metal-organic frameworks (MOFs) are three-dimensional network structures formed through the self-assembly of organic ligands and clusters of metal ions [19]. A distinctive feature of MOFs is that their particle size and morphology can be adjusted by varying the synthesis factors, such as the molar ratio of metal source to organic ligand, the choice of solvent, and the pH level [20,21,22]. The distinct porous structures and tailored compositions of MOFs make them exceptional templates and precursors for synthesizing nanostructured materials through high-temperature pyrolysis. Previous studies have demonstrated that hafnium-based metal-organic frameworks (Hf-MOFs) can be used as precursors for synthesizing HfO_2_/HfC nanoparticles with specific morphologies. For instance, Chen et al. [23] successfully synthesized HfO_2_-embedded porous carbon octahedra (HPCNs) by heating a Hf-MOFs precursor at 750 °C for 0.5 h under a nitrogen atmosphere. Wu et al. [24] synthesized pure HfC nanoparticles with a uniform particle size of 10 nm by pyrolyzing a Hf-MOFs precursor in the air at temperatures up to 2620 °C using a laser as the heating source. However, using a laser as the energy source required precise control of parameters such as the frequency and duration of laser emission, which imposed stricter requirements on equipment and increased the production cost of HfC nanoparticles. This highlights the pressing need for a more convenient, economically viable, and scalable synthesis method for HfC nanoparticles. Regarding related research on synthesizing carbides using amorphous MOF precursors, our previous work successfully prepared an amorphous zirconium-based precursor, which was subsequently pyrolyzed to synthesize high-purity, spherical ZrC nanoparticles [25]. To the best of the author’s knowledge, reports on the direct pyrolysis of Hf-MOFs to synthesize HfC nanoparticles with spherical morphology are still very limited.

Therefore, in this study, a novel amorphous Hf-MOFs was synthesized and then pyrolyzed at 1500 °C to synthesize HfC nanoparticles with spherical morphology, uniform particle size distribution, and high purity. Furthermore, the synthesis process and pyrolysis behavior of the Hf-MOFs were investigated. The effects of the molar ratio of metal source to organic ligand, solvent type, and pyrolysis temperature on the compositions and microstructures of HfC nanoparticles were studied, and the underlying synthesis mechanism was also elucidated. Compared with HfC powders reported in previous literature, the HfC nanoparticles synthesized in this work exhibit superior purity and particle morphology. Furthermore, the resulting ceramics display a high relative density (96.7%) and Vickers hardness (20.2 GPa), underscoring the superiority of the synthesized nanoparticles in fabricating high-quality HfC ceramics.

## 2. Experimental

### 2.1. Raw Materials

Hafnium (VI) tetrachloride (HfCl_4_, 99.5%, Shanghai Aladdin Biochemical Technology Co., Ltd., Shanghai, China), 1.2.4.5-Benzenetetracarboxylic acid (PMA, 98%, Shanghai Macklin Biochemical Co., Ltd., Shanghai, China), Anhydrous ethanol (C_2_H_5_OH, ≥99.8%, Sinopharm Chemical Reagent Co., Ltd., Shanghai, China), Ammonia (NH_4_OH, 25%, Sinopharm Chemical Reagent Co., Ltd., Shanghai, China), Deionized water (DI water) was prepared in-house (Nanjing Tech University, Nanjing, China), Commercially available hafnium carbide (HfC, ≥99%, 200 nm, Beijing Huawei Ruike Chemical Co., Ltd., Beijing, China) were purchased and used without further purification.

### 2.2. Synthesis of HfC Nanoparticles

The required amounts of HfCl_4_ and PMA were weighed accurately and placed into separate vials. Specifically, HfCl_4_ was dissolved in 2.0 mL of DI water for samples HfC-1 to HfC-4. For the subsequent solvent optimization series, HfCl_4_ was dissolved in varying mixtures of DI water and ethanol: 1.5 mL/0.5 mL (HfC-5), 1.0 mL/1.0 mL (HfC-6), 0.5 mL/1.5 mL (HfC-7), and solely 2.0 mL of ethanol (HfC-8). In all cases, PMA was separately dissolved in 2.0 mL of ethanol. In order to ensure complete dissolution, each solution was ultrasonicated for 10 min. The two solutions were then combined and continuously sonicated for another 10 min to achieve uniform mixing. Subsequently, 0.5 mL of NH_4_OH was gradually added to the mixed solution, which was then allowed to stand for 30 min until a gel was formed. The gel was placed in an oven and dried at 120 °C for 18 h. Then, the xerogel was washed with DI water and centrifuged at 8000 rpm for 5 min. The washing process was repeated at least six times to remove residual substances. Finally, the precursor powders were placed in an oven and dried at 120 °C for another 18 h. Specific information of the samples is shown in Table 1.

The as-synthesized precursor powders were placed in a graphite crucible and moved into a vacuum carbon tube furnace (VCF-50-21, Nanjing Boyuntong Instrument Co., Ltd., Nanjing, China). The entire pyrolysis process was conducted under negative pressure (<10 Pa), with a heating rate of 10 °C/min. Initially, the pyrolysis process involved raising the temperature to 800 °C for 3 h. In order to investigate the influence of pyrolysis temperature on the composition and microstructure of the nanoparticles, the temperature was further raised to 1100 °C, 1300 °C, 1400 °C, 1500 °C and 1700 °C, with a holding time of 1 h at each level. Finally, the samples were cooled down to 800 °C at a cooling rate of 10 °C/min, and then naturally cooled down to room temperature.

### 2.3. Synthesis of HfC Ceramics

**Figure 1:**
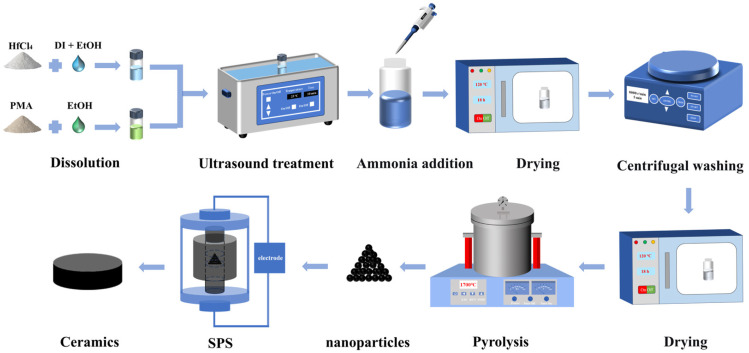
Schematic illustration of the synthesis mechanism of HfC ceramic.

To evaluate the sinterability of the as-synthesized HfC nanoparticles, both the synthesized and commercial HfC powders were consolidated via spark plasma sintering (SPS) under identical conditions without the use of sintering additives. In a typical procedure, 11.5 g of the powder was weighed and loaded into a graphite die (Φ 20 mm) lined with graphite paper. The assembly was then placed into the SPS furnace (S-200D, Shanghai Haoyue Electric Furnace Technology Co., Ltd., Shanghai, China). The sintering process was conducted under a vacuum atmosphere. The samples were heated from room temperature to 1950 °C at a heating rate of 50 °C/min and held for 20 min under a uniaxial pressure of 30 MPa. Subsequently, the pressure was released, and the samples were naturally cooled to room temperature. The schematic of the experiment is depicted in Figure 1.

### 2.4. Characterization

The chemical bonding composition of the as-synthesized precursors was characterized using Fourier-transform infrared spectroscopy (FT-IR, Nexus 670, Nicolet, Madison, WI, USA). The crystalline phase and crystallinity of the pyrolysis samples were analyzed using X-ray diffraction (XRD, SmartLab, Rigaku, Akishima, Tokyo, Japan) with Cu Kα radiation. The size, morphology, and elemental distribution of HfC nanoparticles were examined using a scanning electron microscope (SEM, Regulus 8100, Hitachi, Chiyoda-ku, Tokyo, Japan) and a transmission electron microscope (TEM, Talos F200X G2, FEI, Hillsboro, OR, USA) equipped with energy dispersive spectroscopy (EDS), respectively. The Vickers hardness indentations on the ceramic surfaces were observed using a scanning electron microscope. SEM characterization was carried out at an accelerating voltage of 15 kV with a 30 µm aperture, whereas TEM characterization was performed at 200 kV with a 20 µm aperture. The average particle size was determined by measuring 200 individual HfC nanoparticles from SEM images using Nano Measurer 1.2 software. In order to investigate the thermal stability of the Hf-MOF precursor, HfC-5 was heated to 1500 °C in an argon atmosphere using a simultaneous thermal analyzer (TGA, STA449F3, Netzsch, Selb, Germany) at a heating rate of 10 °C/min. The Brunauer–Emmett–Teller (BET) specific surface area of the HfC nanoparticles was measured using a surface area analyzer (ASAP2460, Micromeritics, Norcross, GA, USA). The ceramic yield was calculated from the mass change of the precursor before and after pyrolysis. Raman spectra of HfC nanoparticles were conducted using a Raman microscope (LabRAM HR800, Horiba, Kyoto, Japan) equipped with a 10× objective lens. The carbon and oxygen contents of the HfC nanoparticles were measured using a sulfur–carbon analyzer (HIR944, Wuxi High-Speed, Wuxi, China) and an oxygen–nitrogen analyzer (EMGA-830, Horiba, Kyoto, Japan), respectively. In order to avoid the influence of residual moisture on the carbon and oxygen content measurements, the HfC nanoparticles (0.5 g) were pre-dried in a vacuum oven (DZF-6020A, Shanghai Jinghong Experimental Equipment Co., Ltd., Shanghai, China) at 80 °C for 12 h. The density and apparent porosity of the as-prepared HfC ceramics were determined based on the Archimedes principle. Vickers hardness was measured using a Vickers hardness tester (HVS-1000Z, Jinan Fengzhi Testing Instrument Co., Ltd., Jinan, China) under an applied load of 1 kgf with a dwell time of 10 s. To obtain a reliable average value, ten indentations were performed at different locations on the ceramic surface.

## 3. Results and Discussion

**Figure 2:**
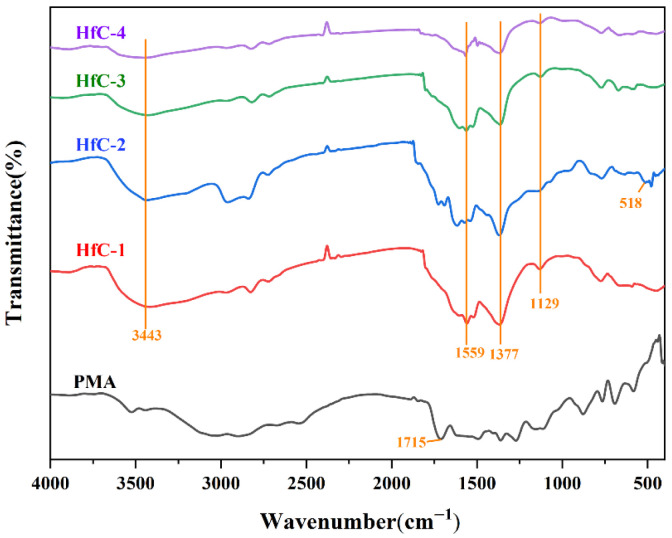
FTIR spectra of Hf-MOFs precursors synthesized with different molar ratios of metal source to organic ligand, along with that of pure PMA for comparison.

Figure 2 shows the FT-IR spectra of Hf-MOFs precursors synthesized with different molar ratios of metal source to organic ligand, together with the spectrum of pure PMA for comparison. A broad absorption band is observed between 3391 and 3451 cm^−1^, corresponding to the O-H stretching vibrations [26], indicating the presence of water molecules in the precursors. Compared with PMA, the C=O stretching vibration peak of the carboxylic acid group (1715 cm^−1^) disappears in the precursors, while new peaks corresponding to the symmetric and asymmetric stretching vibrations of the carboxylate group (COO^−^) appear at around 1371–1382 cm^−1^ and 1554–1563 cm^−1^, respectively [27], which was ascribed to the substitution of H^+^ in PMA by Hf^4+^. Furthermore, all four precursors exhibit similar absorption bands around 1129 cm^−1^, assigned to Hf-O-C bond vibrations [28], providing evidence of coordination between HfCl_4_ and PMA. Additionally, the intensity of the absorption band at this position decreases as the molar ratio of metal source to organic ligand increases. The by-product HCl provided from the hydrolysis lowered the pH value of the aqueous solution, thereby hindering the deprotonation of carboxyl groups and subsequent effective coordination [29]. Notably, an extra vibration peak around 518 cm^−1^ appears in the spectrum of HfC-2. According to previous studies, the vibration peak at this position corresponded to the stretching vibration of the Hf-O-C bond [30], indicating a higher degree of Hf-O-C bond formation in HfC-2.

**Figure 3:**
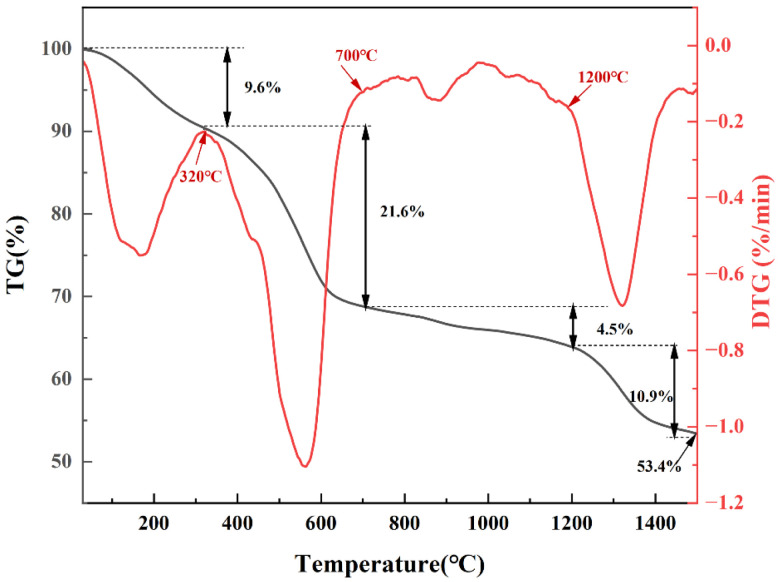
TG-DTG curves of HfC-5.

Figure 3 shows the TG-DTG curves of the Hf-MOFs precursor under an argon atmosphere from 30 °C to 1500 °C, exhibiting a continuous mass loss throughout the entire thermal process. The thermal process of the precursor can be divided into four stages. A mass loss of approximately 9.6% is observed in the first stage over the temperature range of room temperature to 320 °C, which was mainly attributed to the evaporation of adsorbed water [17,18]. This observation was consistent with the presence of water molecules identified by FTIR spectra of Hf-MOFs precursors (Figure 2). Additionally, PMA also underwent volatilization at this stage [25]. A mass loss of 21.6% is observed in the second stage over the temperature range of 320 °C to 700 °C, corresponding to the removal of bound water from HfC-5 and its self-decomposition [17]. The third stage occurs between 700 °C and 1200 °C, during which the TG curve shows a gradual decrease with an additional mass loss of approximately 4.5%. It was due to the release of carbon–oxygen/carbon–hydrogen mixture gases [17,18]. The final stage, from 1200 °C to 1500 °C, exhibits a mass loss of 10.9%, which was attributed to the CTR [31].

**Figure 4:**
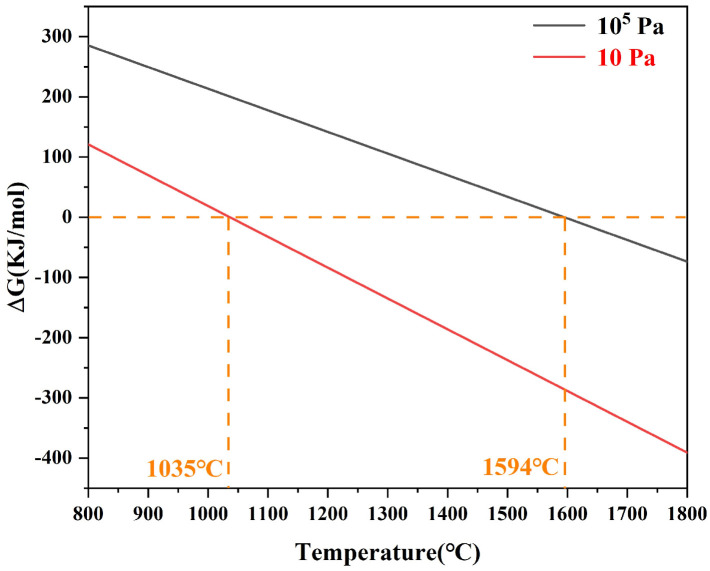
Gibbs free energy variation with temperature for the Equation (1).

Considering the pyrolysis conditions of Hf-MOFs precursor from a thermodynamic perspective, the chemical reaction between *HfO_2_* and graphitic carbon was described by Equation (1), and the change in Gibbs free energy (∆*G_T_*) for this reaction was calculated according to Equation (2).

(1) $$HfO_{2}(s)+3C(s)=HfC(s)+2CO(g)$$

(2) $$\Delta G_{T}=\Delta G_{T}^{0}+RT\cdot \ln{\left(P_{co}∕P_{0}\right)}^{2}$$

where ∆$G_{T}^{0}$ was the change in Gibbs free energy of the reaction under the standard state, *R* was the ideal gas constant (0.008314 kJ/(mol·K)), *T* was the thermodynamic temperature (k), *P_CO_* was CO partial pressure, and *P*_0_ was the standard pressure (1 × 10^5^ Pa). Based on Equation (2) and thermodynamic data, the ∆*G_T_* change as a function of temperature under different *P_CO_* was plotted in Figure 4. The reaction described in Equation (1) is theoretically initiated above 1594 °C under standard conditions (assuming *P_CO_* = 1 × 10^5^ Pa). When the *P_CO_* decreases to 10 Pa, the reaction can start at approximately 1035 °C. Liu et al. [16] reported that HfO_2_ and graphite can be completely converted into HfC through CTR at 1800 °C under vacuum conditions. According to the TG-DTG analysis, considering that the molecularly mixed HfO_2_ and C derived from the Hf-MOFs precursor, and the synthesis of HfC occurred under negative pressure (10 Pa), the synthesis temperature of HfC was expected to be lower. Therefore, a pyrolysis temperature of 1700 °C was selected for subsequent experiments.

**Figure 5:**
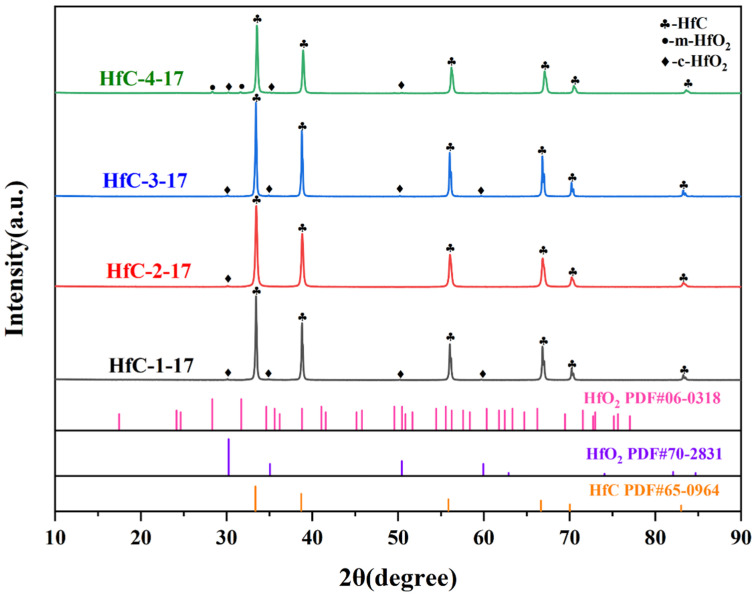
XRD patterns of the pyrolyzed samples (1700 °C) from precursors with varying metal/ligand molar ratios.

The effect of varying molar ratios between the metal source and the organic ligand on the composition of pyrolysis samples at 1700 °C was investigated. As shown in Figure 5, HfC appears as the primary phase in all samples, with minor oxide impurities also present. It was concluded that altering the molar ratio of metal source to organic ligand did not result in the synthesis of pure HfC nanoparticles. The reason was that the dissolution of HfCl_4_ in DI water resulted in the formation of HfCl_2_(OH)_2_. Upon the subsequent addition of NH_4_OH, HfCl_2_(OH)_2_ was further converted into Hf (OH)_4_. During high-temperature pyrolysis, Hf (OH)_4_ decomposed into HfO_2_ and remained in the final samples [32]. The specific reaction processes were as follows:

(3) $$HfCl_{4}(s)+2H_{2}O(l)=HfCl_{2}{\left(OH\right)}_{2}(l)+2HCl(g)$$

(4) $$HfCl_{2}{\left(OH\right)}_{2}(l)+2NH_{4}OH(l)=Hf{\left(OH\right)}_{4}(s)+2NH_{4}Cl(l)$$

(5) $$Hf{\left(OH\right)}_{4}(s)≜HfO_{2}(s)+2H_{2}O(g)$$

**Figure 6:**
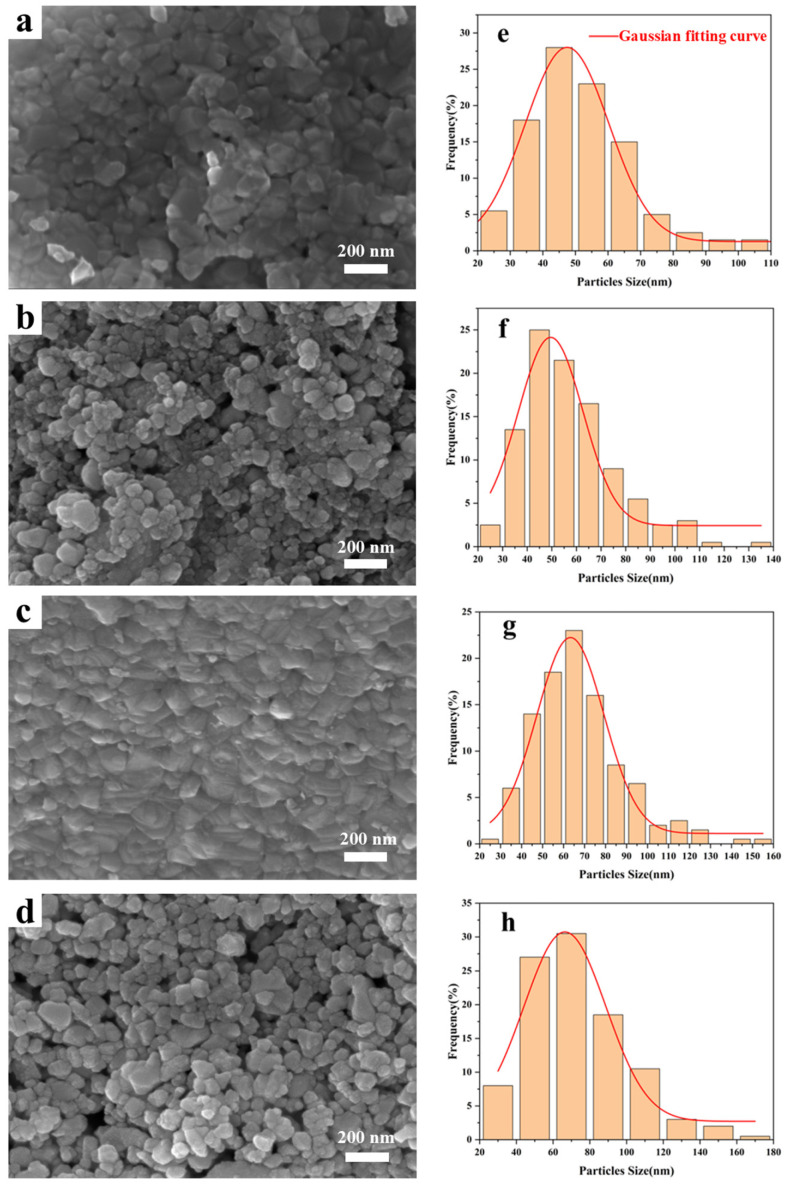
(**a**–**d**) SEM images of HfC nanoparticles, (**e**–**h**) histograms of the particle size distribution. (**a**,**e**) Hf-1-17, (**b**,**f**) Hf-2-17, (**c**,**g**) Hf-3-17, (**d**,**h**) Hf-4-17.

Figure 6 shows the effect of the metal/ligand molar ratios on the morphology of the samples pyrolyzed at 1700 °C. The particle size of HfC grows with an increase of the molar ratio of metal source to organic ligand (Table S1). The presence of HfO_2_ also affected the growth of HfC nanoparticles. Additionally, partial sintering behavior is observed in all four samples, which was caused by the presence of HfO_2_ with low sintering temperature [16]. The yields of the four samples were listed in Table S1, showing minor differences and all exceeding 50%.

In order to obtain phase-pure HfC nanoparticles, it was essential to suppress the hydrolysis of HfCl_4_. Previous studies have mentioned that EtOH can participate in a base deprotonation process and favor the formation of coordination polymer nodes [33]. Figure S1 illustrates the dissolution behavior of HfCl_4_ in different solvents. With the gradual substitution of DI water by EtOH, the solution changed from clear to increasingly turbid. When DI water was completely replaced by EtOH, a milky suspension formed (Figure S1d). The occurrence of the above phenomenon was attributed to the fact that the increase in EtOH content reduced the polarity of the mixed solvent, while HfCl_4_, as a Lewis acid, exhibited poor solubility in solvents with low polarity [34,35]. Moreover, a distinct color change was observed with the addition of EtOH, which was attributed to the alteration of the energy levels of the ligand and metal acceptor orbitals in the mixed solvent, thereby influencing the energy of the Ligand-to-Metal Charge Transfer (LMCT) transition.

**Figure 7:**
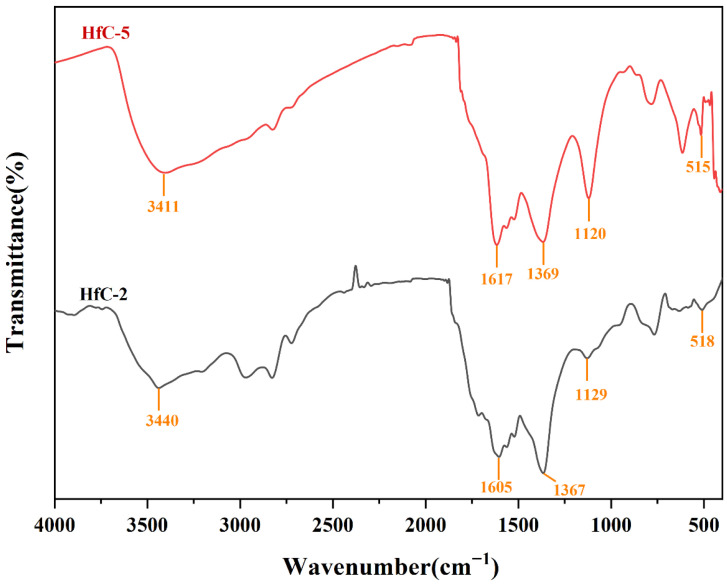
FTIR spectra of HfC-2 and HfC-5.

XRD analysis was performed on HfC-5 (Figure S2). The diffraction pattern of the HfC-5 precursor exhibits two distinct broad diffraction peaks at 2θ = 8° and 30°. This characteristic diffuse scattering pattern provides direct and conclusive evidence for the lack of long-range crystallographic order, strictly confirming its amorphous nature. In order to further investigate the effect of replacing partial DI water with EtOH on the synthesis of HfC nanoparticles, FTIR spectra of HfC-2 and HfC-5 were analyzed. As shown in Figure 7, the O-H stretching vibration absorption band shifts from 3440 cm^−1^ to 3411 cm^−1^, while the symmetric and asymmetric stretching vibrations of the COO^−^ group shift from 1605 and 1367 cm^−1^ to 1617 and 1369 cm^−1^, respectively. Additionally, the Hf-O-C bond vibration peaks shift from 1129 and 518 cm^−1^ to 1120 and 515 cm^−1^. The notably higher intensity of the Hf-O-C vibration peaks in HfC-5 compared to HfC-2 indicated that a greater number of Hf-O-C bonds were formed in the HfC-5. Therefore, replacing 0.5 mL of DI water with EtOH facilitated the coordination between HfCl_4_ and PMA.

**Figure 8:**
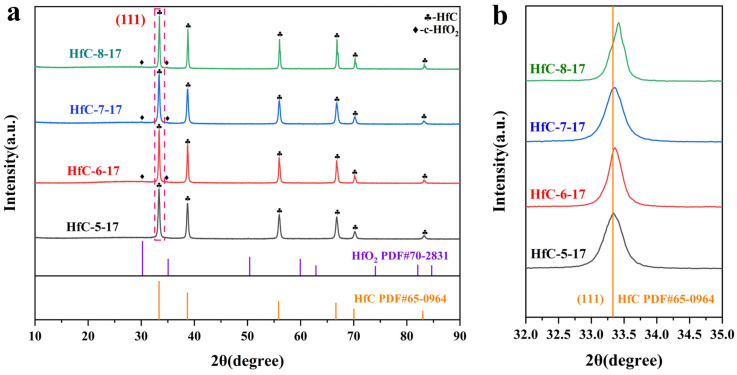
(**a**) XRD patterns of the samples obtained by pyrolyzing, at 1700 °C, the precursors formed by dissolving HfCl_4_ in different solvents. (**b**) Enlarged XRD patterns of the (111) plane of HfC.

The influence of the solvent used to dissolve HfCl_4_ on the composition of the samples pyrolyzed at 1700 °C was investigated. As shown in Figure 8a, diffraction peaks of HfO_2_ are detected in all samples except for HfC-5-17, indicating that introducing a moderate amount of EtOH was crucial for obtaining pure HfC nanoparticles, whereas an excessive amount resulted in the formation of HfO_2_ impurities in the sample. Additionally, HfC-5-17 yields a lower mass output compared to the other three samples (Table 2), demonstrating its higher purity. Figure 8b exhibits the enlargement of the (111) plane of HfC shown in Figure 8a; the diffraction peaks of the other three samples are positioned to the right of HfC-5-17, indicating the partial substitution of carbon atoms (R_C_ = 0.74 Å) by oxygen atoms (R_O_ = 0.67 Å) [36]. The specific changes in lattice parameters were listed in Table 2.

**Figure 9:**
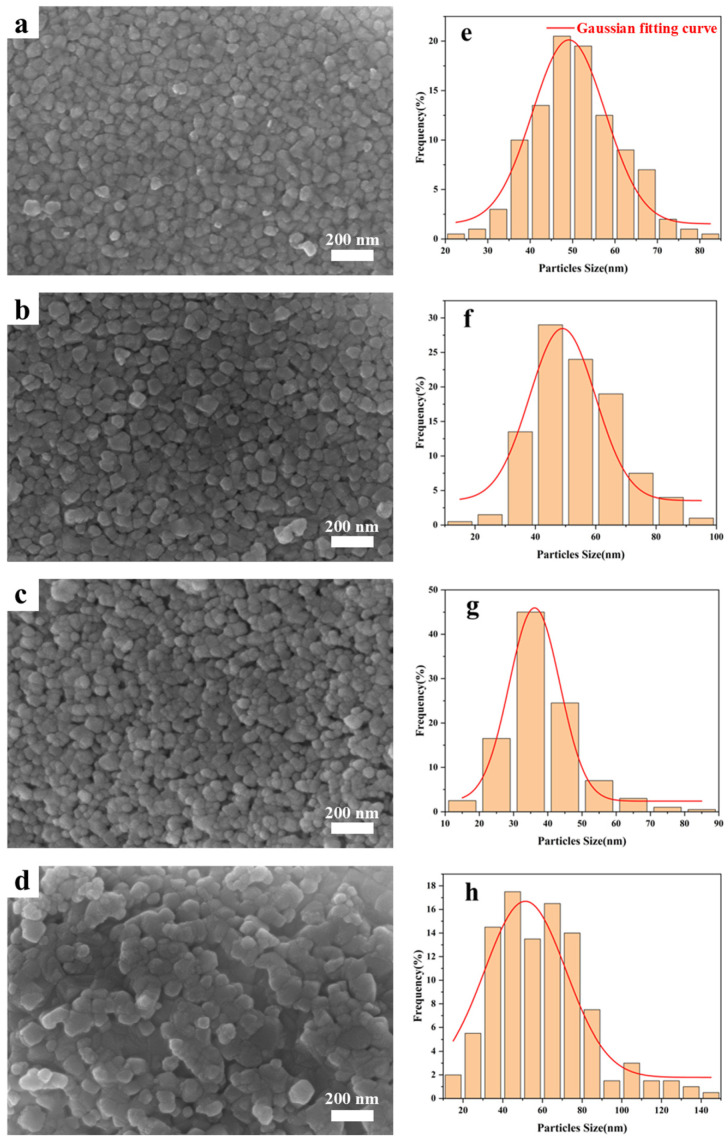
(**a**–**d**) SEM images of HfC nanoparticles, (**e**–**h**) histograms of the particle size distribution. (**a**,**e**) Hf-5-17, (**b**,**f**) Hf-6-17, (**c**,**g**) Hf-7-17, (**d**,**h**) Hf-8-17.

Figure 9 shows the effect of solvent types on the microstructure and particle size distribution of the HfC nanoparticles synthesized at 1700 °C. It can be seen that HfC-5-17 exhibits a uniform size distribution and well-defined spherical morphology. Conversely, the other three samples display irregular shapes and non-uniform sizes. The specific variation in particle size is shown in Table 2. It can be concluded that the morphology and particle size of the synthesized HfC nanoparticles could be tailored by adjusting the solvent composition.

**Figure 10:**
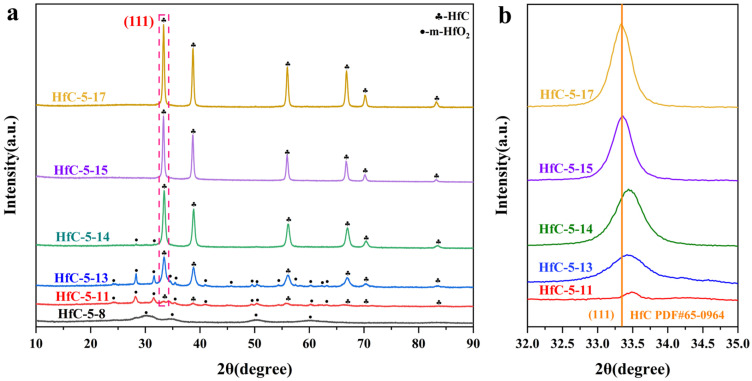
(**a**) XRD patterns of pyrolysis samples with different pyrolysis temperatures, (**b**) enlarged XRD patterns of the (1 1 1) plane of HfC.

Figure 10 presents the XRD patterns of samples heat-treated at various temperatures. As shown in Figure 10a, weak diffraction peaks are observed at 800 °C, corresponding to the formation of monoclinic HfO_2_ (m-HfO_2_). As the temperature increases to 1100 °C, the diffraction peaks corresponding to m-HfO_2_ become more pronounced, and weak HfC peaks begin to appear, indicating the initiation of the CTR between carbon and HfO_2_. While thermodynamically feasible at ~1035 °C, significant HfC formation requires ≥1100 °C due to kinetic limitations. Initially, sluggish atomic diffusion produces ultra-small nuclei below conventional XRD detection limits. Furthermore, intermediate layers at the HfO_2_/C interface create physical diffusion barriers. Finally, breaking the strong Hf-O bonds and removing lattice oxygen requires substantial thermal energy, thereby significantly elevating the actual macroscopic conversion temperature. As the temperature increases from 1300 °C to 1400 °C, HfC progressively emerges as the dominant phase. The continuous intensification of HfC peaks and the concurrent consumption of residual m-HfO_2_ indicate an active, yet incomplete, carbothermal reduction (CTR) process. When the temperature reaches or exceeds 1500 °C, no oxides diffraction peak can be observed. As shown in Figure 10b, the diffraction peaks of HfC gradually shift toward lower angles with increasing pyrolysis temperature and remain unchanged once the temperature reaches 1700 °C. The oxygen atoms in HfC were gradually removed as the pyrolysis temperature increased, and the lattice parameters increased accordingly (Table 3), resulting in an improvement in the purity of the HfC nanoparticles. Additionally, as the pyrolysis temperature increases, the yield of the samples decreases correspondingly (Table 3), further confirming that the rise in temperature facilitated the removal of oxygen impurities.

**Figure 11:**
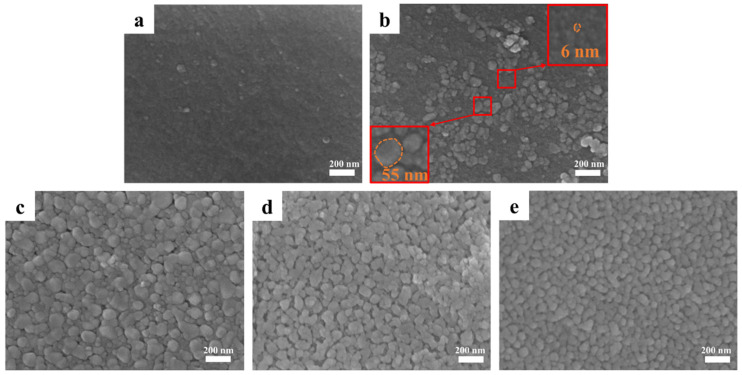
SEM images of the pyrolysis products. (**a**) HfC-5-8, (**b**) HfC-5-11, (**c**) HfC-5-13, (**d**) HfC-5-14, (**e**) HfC-5-15.

Figure 11 shows the SEM characterization of HfC obtained at different pyrolysis temperatures, revealing the evolution of the sample microstructure with increasing temperature. The particle microstructure of HfC-5-8 exhibits the aggregated block characteristic. According to the EDS results (Figure S3 and Table S2), the pyrolysis sample at 800 °C was composed of HfO_2_ and carbon. The presence of carbon inhibited the growth of HfO_2_ particles, thereby inevitably leading to their agglomeration [37]. HfC-5-11 exhibits two distinctly different particle sizes (~55 nm and ~6 nm). The morphological transformation was associated with the progressive conversion of HfO_2_ to HfC [38]. According to the EDS results (Figure S4 and Table S3), the large and small particles were related to HfO_2_ wrapped by carbon and HfC, respectively, which was consistent with the XRD results (Figure 10a). In the intermediate temperature range (1300–1400 °C), the newly nucleated HfC nanoparticles exhibit a non-uniform, fine size distribution and a strong tendency to form large aggregates. This severe agglomeration is primarily driven by the inherently high surface energy of the ultra-small crystallites generated during the incomplete CTR process. HfC-5-15 and HfC-5-17 are composed of isolated and uniformly sized spherical HfC nanoparticles. The particle sizes, measured from the SEM images (Figure 9a and Figure 11e), are 44.30 ± 9.63 nm for HfC-5-15 and 51.17 ± 10.39 nm for HfC-5-17, respectively, accompanied by a corresponding decrease in specific surface area from 106.66 m^2^/g to 60.91 m^2^/g.

**Figure 12:**
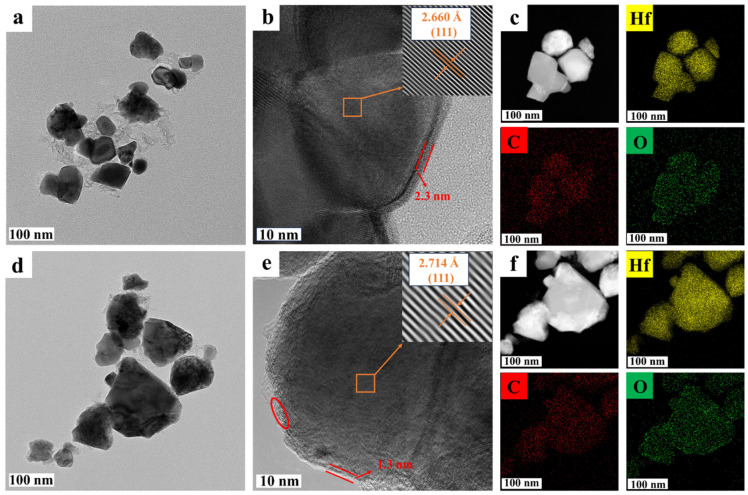
LRTEM images, HRTEM images, and EDS mapping of HfC-5-15 (**a**–**c**) and HfC-5-17 (**d**–**f**), respectively.

To further investigate the microstructure of HfC-5-15 and HfC-5-17, TEM characterizations were conducted. As shown in Figure 12a,d, the average particle sizes of the HfC-5-15 and HfC-5-17 nanoparticles are 40.68 ± 15.3 nm and 53.45 ± 10.5 nm, respectively, which was consistent with the SEM results (Figure 9a and Figure 11e). Additionally, residual carbon is observed in HfC-5-15, whereas no obvious residual carbon is found in HfC-5-17. To further determine the carbon contents of HfC-5-15 and HfC-5-17, sulfur–carbon analysis was conducted. The results showed that the carbon contents of HfC-5-15 and HfC-5-17 were 11.21% and 6.42%, respectively, suggesting that increasing the pyrolysis temperature effectively removed the excess carbon. Notably, the presence of an amorphous layer is observed in HfC-5-15 and HfC-5-17 nanoparticles (Figure 12b,e). Moreover, EDS analysis reveals a uniform distribution of Hf, O, and C elements within the HfC nanoparticles (Figure 12c,f), although no HfO_2_ phase is detected in the XRD patterns (Figure 10a). The residual oxygen present in the surface layer of the particles is the primary reason for the detected oxygen. The formation of an amorphous coating layer was explained through the gas–solid reaction theory [36]. HfO and CO gases, generated by the destabilization of HfO_2_ and C during the temperature increase, condensed to form an amorphous layer on the surface of HfC, which is primarily composed of an oxygen-rich Hf-O-C amorphous phase [17,39]. As the temperature increases from 1500 °C to 1700 °C, the thickness of the coating layer on the HfC nanoparticles decreases from approximately 2.3 nm to 1.3 nm, and Hf-O-C nuclei are also observed in the amorphous carbon layer at 1700 °C, indicating that HfC_x_O_y_ was further converted into HfC. The specific reaction processes are as follows:

(6) $$HfO_{2}(s)\to HfO(g)+\frac{1}{2}O_{2}(g)$$

(7) $$C(s)+\frac{1}{2}O_{2}(g)\to CO(g)$$

(8) $$HfO_{2}(s)+CO(g)\to HfO(g)+CO_{2}(g)$$

(9) $$C(s)+CO_{2}(g)\to 2\;CO(g)$$

(10) $$HfO(g)+CO(g)\to HfC_{x}O_{y}(s)+CO_{2}(g)$$

(11) $$HfC_{x}O_{y}(s)+CO(g)\to HfC_{1-a}(s)+CO_{2}(g)$$

HRTEM images (Figure 12b,e) confirm that HfC-5-15 and HfC-5-17 have lattice fringes with interplanar spacings of 0.2660 nm and 0.2714 nm, respectively, which correspond to the (111) plane of HfC.

**Figure 13:**
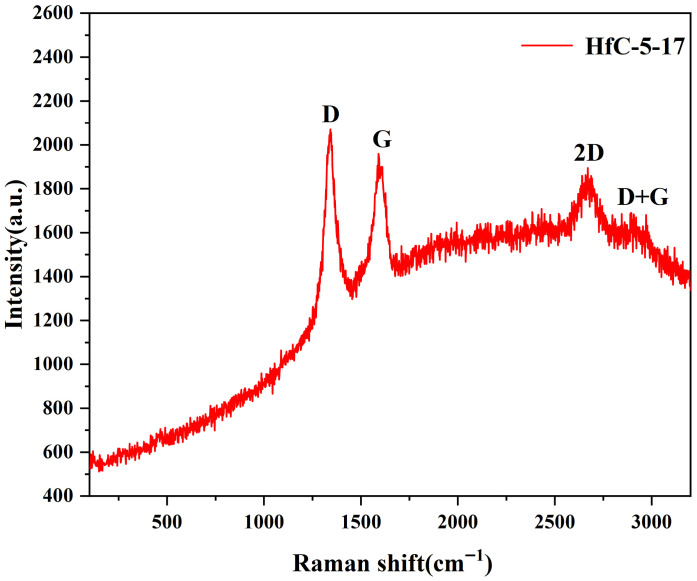
Raman spectroscopy of HfC-5-17.

Raman spectroscopy was performed over a broadened wavenumber range of 100–3200 cm^−1^ to comprehensively characterize both the HfC crystal lattice and the structural features of the carbon. As shown in Figure 13, no distinct Raman peaks are observed in the low-frequency region (100–1000 cm^−1^). Since perfectly stoichiometric cubic HfC (with an Fm-3m rock-salt structure) is first-order Raman inactive due to its inversion symmetry, the absence of defect-induced phonon modes experimentally confirms the high crystallinity and near-perfect stoichiometry of the synthesized HfC nanoparticles. Two pronounced Raman peaks appear at approximately 1350 cm^−1^ (D band) and 1580 cm^−1^ (G band), confirming the presence of sp^2^-hybridized carbon in the HfC nanoparticles [40]. The G band corresponds to the ideal graphitic lattice, while the D band is associated with disordered graphitic structures [41]. The I_D_/I_G_ intensity ratios of HfC-5-17 and HfC-5-15 (Figure S5) were 1.46 and 1.75, respectively, indicating a highly disordered structure. Therefore, the disordered carbon was dominant in both HfC-5-15 and HfC-5-17. The 2D band was observed at approximately 2600–2700 cm^−1^, along with a weak D+G band at 2800 cm^−1^. The 2D band is a characteristic second-order overtone of the carbon lattice, originating from a double-resonance process involving two phonons of opposite wavevectors. The clear presence of these second-order features provides compelling evidence for the development of an ordered sp^2^ carbon network.

**Figure 14:**
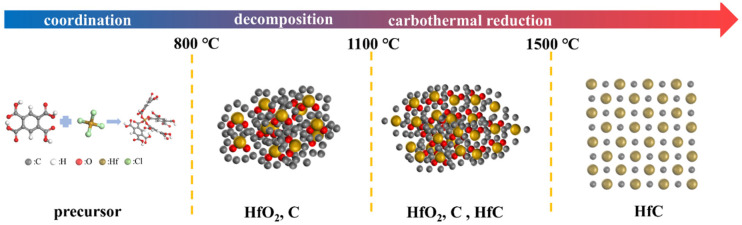
Schematic illustration of the formation mechanism of HfC nanoparticles.

Based on the above experimental observations and discussion, the synthesis process of HfC nanoparticles could be divided into two main stages, and the corresponding formation mechanism was illustrated in Figure 14.

(1) Coordination reaction: During the initial coordination stage, HfCl_4_ reacted with PMA through a chelation-coordination process. The carboxyl groups (-COOH) of PMA underwent deprotonation, generating negatively charged carboxylate ions (-COO^−^). These oxygen atoms possessed strong electron-donating ability and could effectively coordinate with the empty orbitals of Hf^4+^ ions through oxygen–metal interactions. As a result, stable Hf-O-C coordination bonds were formed, leading to the construction of an Hf-organic network. This coordination framework ensured the homogeneous distribution of Hf and C at the molecular level, which was favorable for the subsequent formation of uniformly sized HfC nanoparticles during pyrolysis.
(2) Pyrolysis reaction: The Hf-MOFs precursor underwent pyrolysis, during which the organic ligands were carbonized to form amorphous carbon, while the Hf-O-C bonds were disrupted, resulting in the formation of HfO_2_. Consequently, HfO_2_ and carbon were uniformly mixed at the nanoscale, providing a highly reactive interface for the subsequent CTR. Subsequently, the formation of HfC nanoparticles initiated at around 1100 °C, which was in close agreement with the critical temperature (1035 °C) predicted by the Gibbs free energy calculation. Finally, high-purity, spherical HfC nanoparticles with uniform size distribution were synthesized at 1500 °C.

To explicitly highlight the differences among various synthesis strategies, Table 4 presents a broad literature comparison, further demonstrating the distinct advantages of our novel MOF-derived route in terms of reduced pyrolysis temperature and morphological control. Specifically, the MOF-derived ceramic method produced spherical HfC nanoparticles with the finest reported particle size at the lowest pyrolysis temperature reported to date. While pyrolysis at 1500 °C resulted in a phase-pure HfC identified by XRD, the sample contained slightly higher contents of residual carbon and oxygen. However, raising the temperature to 1700 °C effectively mitigated the carbon impurity, bringing the measured carbon content (6.42%) remarkably close to the theoretical value of 6.31%. This precise stoichiometric control represented a significant benefit for powder synthesis. The persistent, though slight, oxygen content in the 1700 °C sample was likely a consequence of its minimal particle size, which increased the specific surface area and propensity for surface oxidation. Consequently, this MOF-derived synthesis strategy offers a promising and feasible pathway for the manufacturing of high-quality UHTC powders, ceramics and composites.

To rigorously evaluate the sintering activity of the synthesized powders, a strictly controlled parallel experiment was conducted by sintering both HfC-5-17 and commercial HfC powders under identical conditions (SPS, 1950 °C/30 MPa/20 min). Table 5 summarizes the sintering performance data obtained in this work alongside reported values for carbide ceramics from the literature. The results indicate that the HfC-5-17 ceramics achieved a relative density of 96.7% and an apparent porosity of 0.5%, whereas the commercial HfC ceramics exhibited values of 95.8% and 0.7%, respectively. This discrepancy was primarily attributed to the difference in particle size: the commercial HfC powder (~230 nm) was significantly coarser than the HfC-5-17 powder (~50 nm). Consequently, the specific surface area of the commercial powder (4.02 m^2^/g) was much lower than that of the HfC-5-17 powder (60.91 m^2^/g). It is well established that a larger specific surface area provides a greater thermodynamic driving force for sintering. Furthermore, the commercial HfC powder exhibited an irregular morphology with noticeable agglomeration (see Figure S6), which tended to result in residual pores during sintering, thereby limiting the final density [44]. In contrast, the spherical morphology of the HfC-5-17 nanoparticles enhanced powder flowability and packing density, effectively accelerating the densification process.

**Figure 15:**
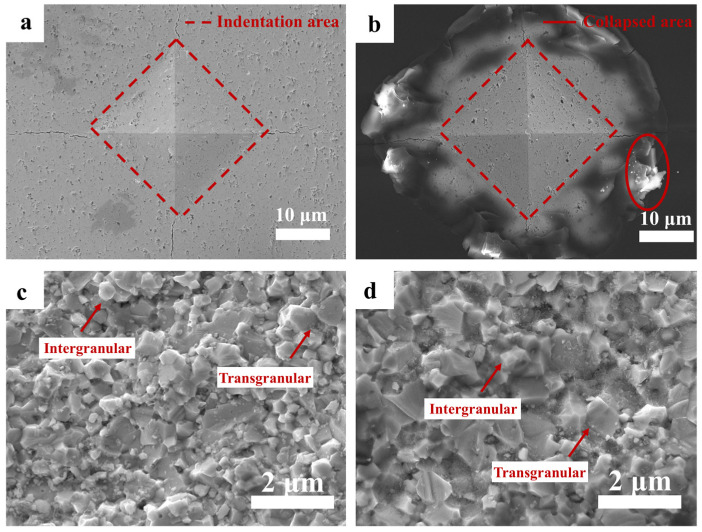
SEM images of Vickers hardness indentations and cross-section of HfC-5-17 ceramic (**a**,**c**) and commercial HfC ceramic (**b**,**d**), respectively.

Figure 15a,b displays the SEM images of Vickers hardness indentations on the ceramic surfaces. The measured Vickers hardness values for the HfC-5-17 and commercial HfC ceramics were 20.2 GPa and 17.8 GPa, respectively. For the HfC-5-17 ceramic, a clear indentation profile with shorter diagonals was observed; the cracks induced by the indentation propagated radially outward, while the surrounding microstructure remained intact. In contrast, the commercial HfC ceramic exhibited significant spalling and collapse around the indentation area. As shown in Table 5, compared to other methods for preparing HfC/ZrC ceramics, the HfC ceramics prepared in this study were sintered at a lower temperature and pressure, yet they still exhibited superior hardness. This further corroborates the significant advantages of the HfC-5-17 nanoparticles in fabricating high-quality ceramics. Figure 15c,d presents the cross-sectional SEM microstructures of the ceramics. Both the HfC-5-17 and commercial HfC ceramics exhibited a mixed fracture mode comprising both transgranular and intergranular fractures, as evidenced by the presence of cleaved grain facets and intact grains. This fracture behavior reflects the competition between grain boundary bonding strength and intragranular cohesive strength.

**Figure 16:**
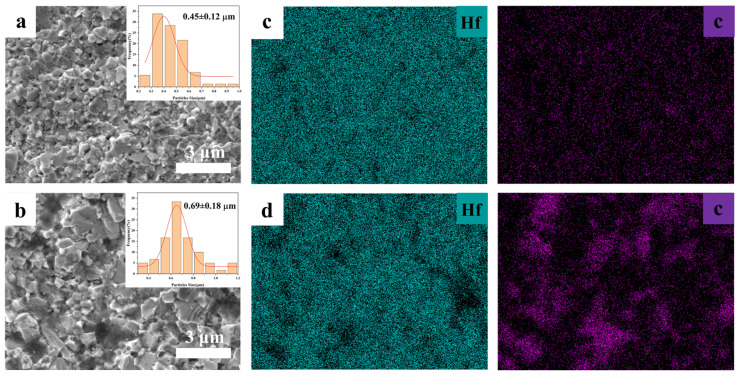
SEM images of cross-section and EDS mapping of HfC-5-17 ceramic (**a**,**c**) and commercial HfC ceramic (**b**,**d**), respectively.

Figure 16 presents the cross-sectional SEM images and corresponding elemental mapping of the HfC-5-17 and commercial HfC ceramics. The average grain sizes of the HfC-5-17 and commercial HfC ceramics were determined to be 0.45 ± 0.12 μm and 0.69 ± 0.18 μm, respectively. The sintered HfC-5-17 ceramics exhibited a fine and uniform microstructure with tightly bonded grains. In contrast, the commercial HfC grains showed evident coarsening with a broader size distribution and distinct angular morphologies. As revealed by the elemental mapping, significant elemental segregation was induced in the commercial HfC sintered body, which can be attributed to the agglomeration and packing defects caused by the irregular morphology of the starting powder. Conversely, the HfC-5-17 nanoparticles, benefiting from their excellent dispersibility and packing characteristics, effectively shortened the solid-state diffusion path, thereby achieving a highly homogeneous distribution of Hf and C elements at the microscale.

## 4. Conclusions

In this paper, a novel amorphous Hf-MOFs precursor was successfully synthesized using HfCl_4_ and PMA as raw materials, with EtOH and DI water serving as mixed solvents. Experimental results showed that by employing a metal/ligand molar ratio of 1.5 and introducing 0.5 mL of EtOH, the resulting precursor could be converted into high-purity, spherical HfC nanoparticles with uniform particle size (44.30 ± 9.63 nm) via one-step pyrolysis at 1500 °C under vacuum. Compared with dissolving HfCl_4_ in pure DI water, the introduction of EtOH into the solvent promoted the formation of more Hf-O-C bonds, resulting in HfC nanoparticles with higher purity and more regular morphology. The synthesis temperature for HfC nanoparticles via the MOF-derived method is lower than that reported for other preparation methods in the literature. The HfC nanoparticles synthesized at 1700 °C exhibited extremely low oxygen and carbon contents (0.76%, 6.42%), with a slight increase in particle size (51.17 ± 10.39 nm) while maintaining a regular morphology. High-quality HfC ceramics were fabricated using the synthesized HfC powders via SPS at 1950 °C under a pressure of 30 MPa for 20 min. The resulting ceramics exhibited a relative density of 96.7% and a Vickers hardness of 20.2 GPa, which are superior to the values obtained for the commercial HfC counterparts (95.8% and 17.8 GPa, respectively).

This work not only successfully synthesized spherical HfC nanoparticles at relatively low temperatures, but also elucidated their formation mechanism. The strategy offers a practical alternative to conventional high-temperature methods and holds promise for extending to other spherical UHTC systems. The resulting powders are particularly suited for improving infiltration and processability in UHTCMCs fabrication, highlighting both the novelty and application potential of the approach.

## Figures and Tables

**Table 1:** The specific information of the samples.

HfCl_4_ (mg)	PMA (mg)	Molar Ratio	DI Water (mL)	C_2_H_5_OH (mL)	NH_4_OH (mL)	Precursor	Pyrolytic Temperature (°C)	HfC
320	254	1:1	2	2	0.5	HfC-1	1700	HfC-1-17
480	254	1.5:1	2	2	0.5	HfC-2	1700	HfC-2-17
640	254	2:1	2	2	0.5	HfC-3	1700	HfC-3-17
800	254	2.5:1	2	2	0.5	HfC-4	1700	HfC-4-17
480	254	1.5:1	1.5	0.5 + 2	0.5	HfC-5	1700	HfC-5-17
480	254	1.5:1	1	1 + 2	0.5	HfC-6	1700	HfC-6-17
480	254	1.5:1	0.5	1.5 + 2	0.5	HfC-7	1700	HfC-7-17
480	254	1.5:1	-	2 + 2	0.5	HfC-8	1700	HfC-8-17
480	254	1.5:1	1.5	0.5 + 2	0.5	HfC-5	800	HfC-5-8
480	254	1.5:1	1.5	0.5 + 2	0.5	HfC-5	1100	HfC-5-11
480	254	1.5:1	1.5	0.5 + 2	0.5	HfC-5	1300	HfC-5-13
480	254	1.5:1	1.5	0.5 + 2	0.5	HfC-5	1400	HfC-5-14
480	254	1.5:1	1.5	0.5 + 2	0.5	HfC-5	1500	HfC-5-15

**Table 2:** Properties of HfC nanoparticles synthesized using different solvent types.

Sample	Yield (%)	Lattice Parameters (Å)	Particle Size (nm)
HfC-5-17	50.7	4.6458	51.17 ± 10.39
HfC-6-17	52.1	4.6448	54.19 ± 14.17
HfC-7-17	51.5	4.6455	38.32 ± 10.76
HfC-8-17	51.9	4.6398	60.17 ± 24.95

**Table 3:** Yields and lattice parameters of HfC nanoparticles obtained at different temperatures.

Sample	Yield (%)	Lattice Parameters (Å)
HfC-5-8	68.5	-
HfC-5-11	65.1	-
HfC-5-13	60.6	4.6398
HfC-5-14	54.6	4.6421
HfC-5-15	52.7	4.6450

**Table 4:** Summary of the information HfC nanoparticles prepared by different synthesis methods.

Method	Pyrolysis Temperature (°C)	Carbon Content (%)	Oxygen Content (%)	Particle Size (nm)	Morphology	Ref.
CTR	1800	6.74	0.72	225–380	Irregular	[16]
Sol-gel	1700	-	0.69	500	Irregular	[42]
PDC	1600	-	0.64	73	Equiaxial	[17]
PDC	1550	-	0.97	350	Near-Spherical	[43]
MOFs	1500	11.21	1.28	44	Spherical	Current work
MOFs	1700	6.42	0.76	51	Spherical	Current work

**Table 5:** Summary of sintering parameters and properties of different carbide ceramics.

Sample	Temperature (°C)	Pressure (MPa)	Particle Size (μm)	Relative Density (%)	Grain Size (μm)	Vickers Hardness (GPa)
SPS-HfC [45]	1850	40	0.3	70	>2	0.9 ± 0.1
HP-HfC [46]	1900	30	0.7	89	20–30	5.79 ± 0.61
RSPS-HfC [47]	1900	60	1	92.3	<5	15.9 ± 0.5
SPS-HfC [39]	2000	80	0.11	99.1	6.7 ± 0.7	19.6
SPS-ZrC [48]	2000	40	3.5	94	6.50 ± 0.44	16.49 ± 0.21
HfC-5-17	1950	30	0.05	96.7	0.45 ± 0.12	20.2
Commercial HfC	1950	30	0.23	95.8	0.69 ± 0.18	17.8

## Data Availability

The original contributions presented in this study are included in the article/Supplementary Material. Further inquiries can be directed to the corresponding authors.

## Supplementary Materials

The following supporting information can be downloaded at: https://www.mdpi.com/article/10.3390/ma19091754/s1, Table S1: Yield and particle size of HfC with different molar ratios of metal source to organic ligand; Table S2: EDS analysis of HfC-5-8; Table S3: EDS analysis of HfC-5-11; Figure S1: (a–d) Dissolution behavior of HfCl_4_ in different solvents, (a) 1.5 mL DI water + 0.5 mL EtOH, (b) 1 mL DI water + 1 mL EtOH, (c) 0.5 mL DI water + 1.5 mL EtOH, (d) 2 mL EtOH; Figure S2: XRD pattern of the HfC-5 precursor; Figure S3: EDS elemental mapping of HfC-5-8 nanoparticles; Figure S4: SEM image of HfC-5-11; Figure S5: Raman spectroscopy of HfC-5-15; Figure S6: The SEM image of commercial HfC ceramic powders.
