# Supplementary material for: High-Purity, Uniform, and Spherical Hafnium Carbide Nanoparticles Derived from a Novel Amorphous Hafnium-Based Metal–Organic Framework Precursor for the Preparation of High-Performance Ceramics
Source: Materials (Basel). 2026 Apr 24;19(9):1754. doi: 10.3390/ma19091754 (PMC13165069; doi:10.3390/ma19091754)
Supplement: Supplementary file 1 [file materials-19-01754-s001.zip › materials-4240506-supplementary.pdf]

High-Purity, Uniform, and Spherical Hafnium Carbide Nanoparticles Derived from a Novel Amorphous Hafnium-Based Metal–Organic Framework Precursor for the Preparation of High-Performance Ceramics

Hongzhi Cheng[a,b,1], Jian Gu[a,b,1*], Siyuan Kan[a], Ran Xie[a], Quan Li[a,*], Sinuo Zhang[c,*], Junyang Jin[a,b,*], Yang Wang[a,b], Jian Yang[a,b], Chang-An Wang[d]

[a]College of Materials Science and Engineering, Nanjing Tech University, Nanjing 211816, China

[b]Jiangsu Collaborative Innovation Center for Advanced Inorganic Function Composites, Nanjing 211816, China

[c]Advanced Bio and Healthcare Materials Research Division, Korea Institute of Materials Science (KIMS), Changwon 51508, Republic of Korea

[d]State Key Lab of New Ceramics and Fine Processing, School of Materials Science and Engineering, Tsinghua University, Beijing 100084, China

1: These two authors contributed equally to this work.

[*]Corresponding author.

Jian Gu E-mail address: gujianhll1234@gmail.com

Quan Li E-mail address: quan@njtech.edu.cn

Sinuo Zhang E-mail address: zhsinuo@outlook.com

Junyang Jin E-mail address: jjy@njtech.edu.cn

The following are the supplementary material to this article:

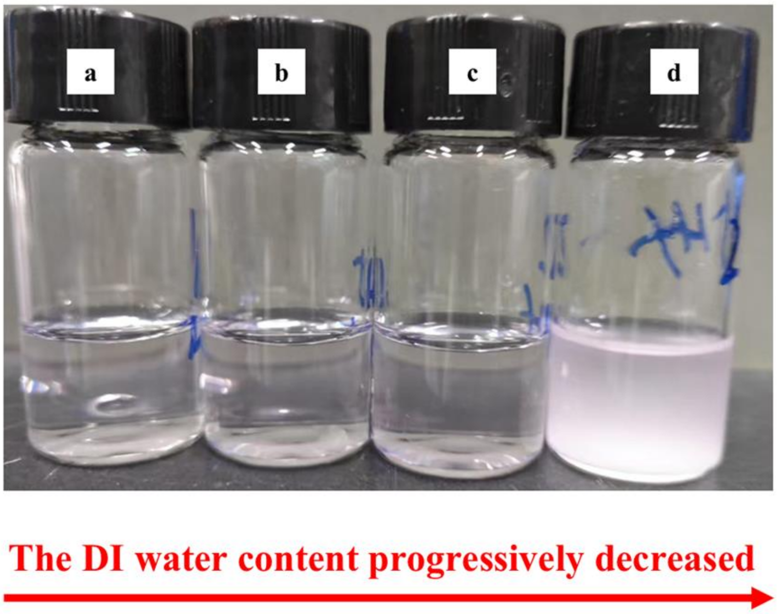

Figure S1. (a-d) Dissolution behavior of $HfCl_4$ in different solvents, (a) 1.5 ml DI water + 0.5 ml EtOH, (b) 1 ml DI water + 1 ml EtOH, (c) 0.5 ml DI water + 1.5 ml EtOH, (d) 2 ml EtOH.

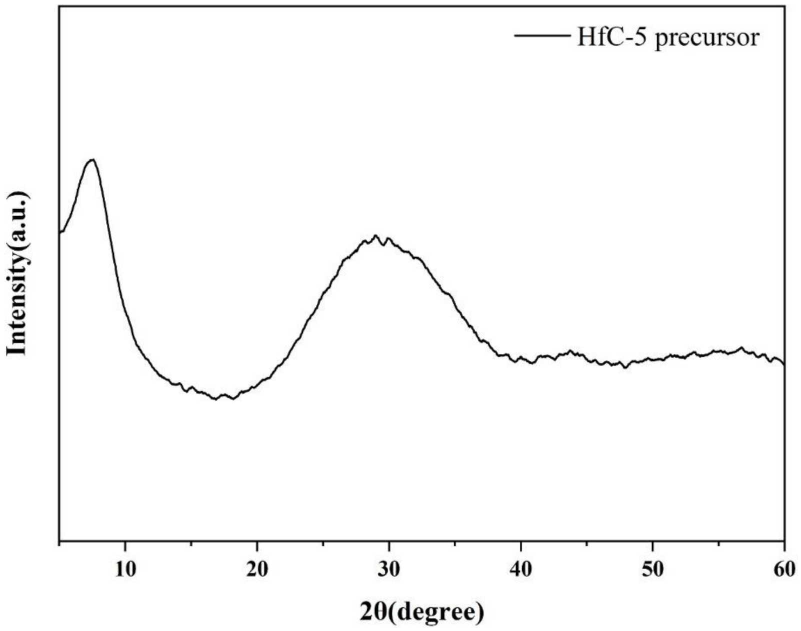

Figure S2. XRD pattern of the HfC-5 precursor.

Table S1. Yield and particle size of HfC with different molar ratios of metal source to organic ligand.

Sample	Yield (%)	Particle size (nm)
HfC-1-17	53.3	51.49±16.04
HfC-2-17	51.9	57.50±18.68
HfC-3-17	54.5	67.15±20.94
HfC-4-17	57.6	72.96±26.47

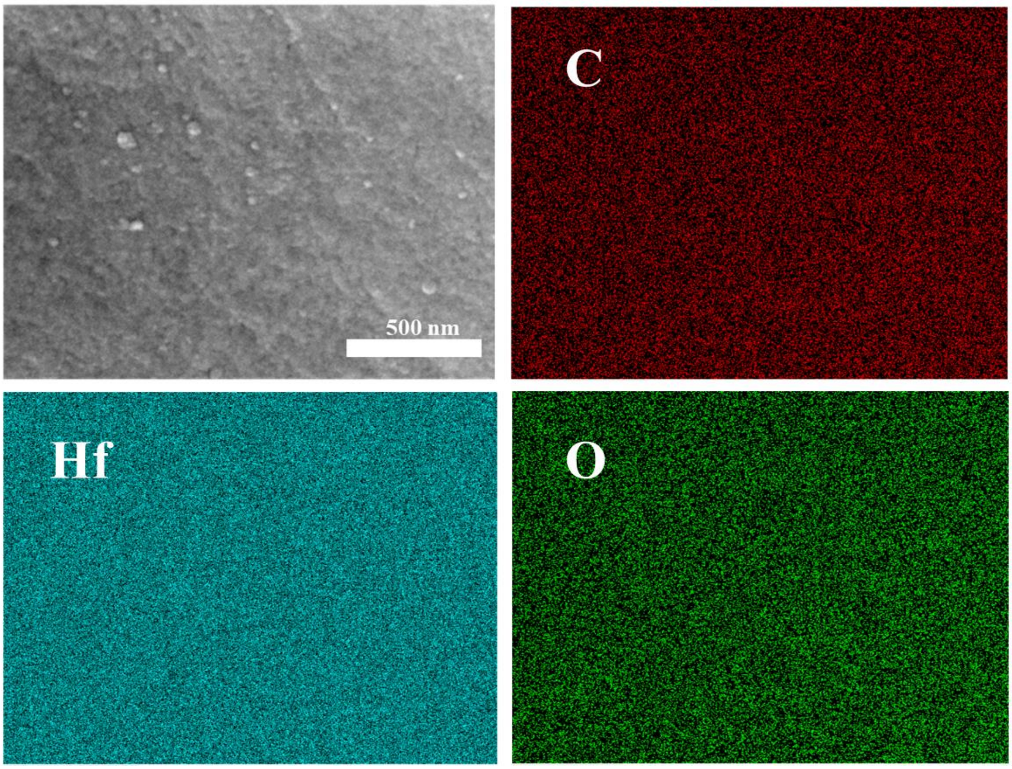

Figure S3. EDS elemental mapping of HfC-5-8 nanoparticles.

Table S2. EDS analysis of HfC-5-8.

Hf (wt%)	C (wt%)	O (wt%)
72.08	17.76	10.16

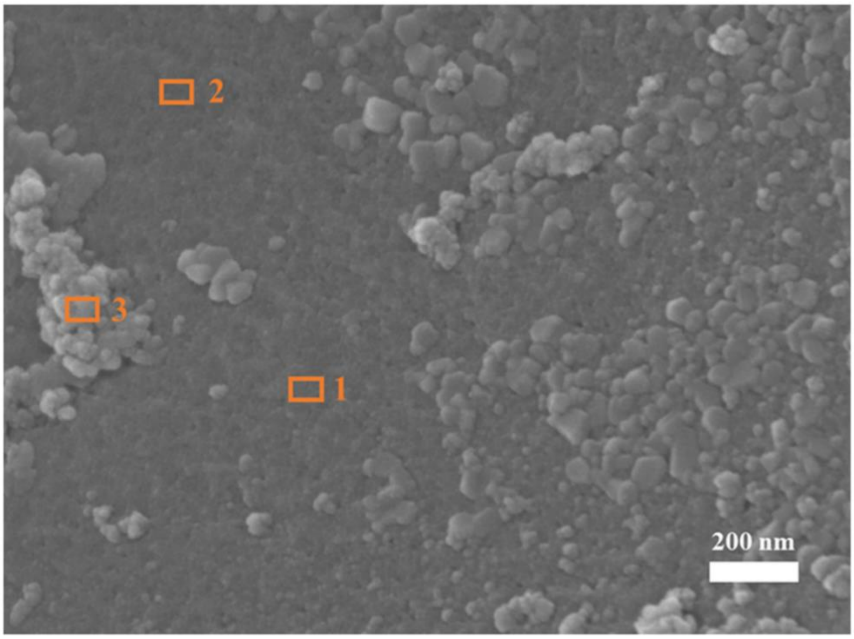

Figure S4. SEM image of HfC-5-11.

Table S3. EDS analysis of HfC-5-11.

Position Fig. S2	Hf (wt%)	C (wt%)	O (wt%)
1	79.38	13.13	7.49
2	77.90	15.22	6.88
3	72.5	18.65	9.30

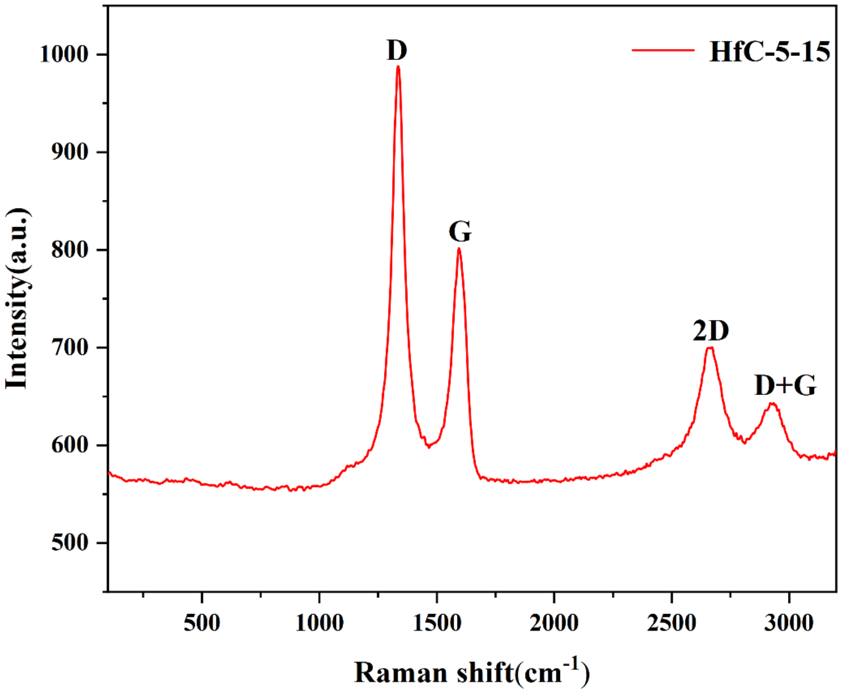

Figure S5. Raman spectroscopy of HfC-5-15.

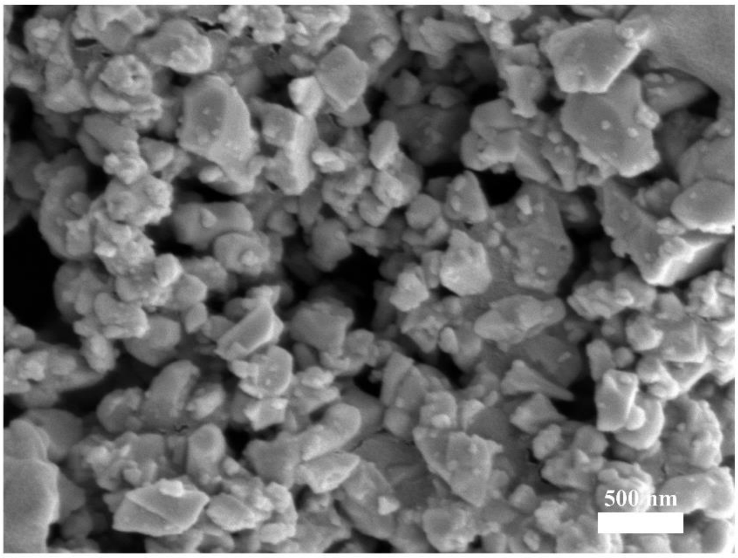

Figure S6. The SEM image of commercial HfC ceramic powders.
